# Expanding anchored hybrid enrichment to resolve both deep and shallow relationships within the spider tree of life

**DOI:** 10.1186/s12862-016-0769-y

**Published:** 2016-10-13

**Authors:** Chris A. Hamilton, Alan R. Lemmon, Emily Moriarty Lemmon, Jason E. Bond

**Affiliations:** 1 Department of Biological Sciences, Auburn University & Auburn University Museum of Natural History, Auburn, AL USA; 2 Department of Scientific Computing, Florida State University, Tallahassee, FL USA; 3 Department of Biological Science, Florida State University, Tallahassee, FL USA

**Keywords:** Anchored Hybrid Enrichment, Anchored phylogenomics, Targeted sequence capture, Conserved regions, Ultraconserved elements, Araneae, Mygalomorphae, Arachnida

## Abstract

**Background:**

Despite considerable effort, progress in spider molecular systematics has lagged behind many other comparable arthropod groups, thereby hindering family-level resolution, classification, and testing of important macroevolutionary hypotheses. Recently, alternative targeted sequence capture techniques have provided molecular systematics a powerful tool for resolving relationships across the Tree of Life. One of these approaches, Anchored Hybrid Enrichment (AHE), is designed to recover hundreds of unique orthologous loci from across the genome, for resolving both shallow and deep-scale evolutionary relationships within non-model systems. Herein we present a modification of the AHE approach that expands its use for application in spiders, with a particular emphasis on the infraorder Mygalomorphae.

**Results:**

Our aim was to design a set of probes that effectively capture loci informative at a diversity of phylogenetic timescales. Following identification of putative arthropod-wide loci, we utilized homologous transcriptome sequences from 17 species across all spiders to identify exon boundaries. Conserved regions with variable flanking regions were then sought across the tick genome, three published araneomorph spider genomes, and raw genomic reads of two mygalomorph taxa. Following development of the 585 target loci in the Spider Probe Kit, we applied AHE across three taxonomic depths to evaluate performance: deep-level spider family relationships (33 taxa, 327 loci); family and generic relationships within the mygalomorph family Euctenizidae (25 taxa, 403 loci); and species relationships in the North American tarantula genus *Aphonopelma* (83 taxa, 581 loci). At the deepest level, all three major spider lineages (the Mesothelae, Mygalomorphae, and Araneomorphae) were supported with high bootstrap support. Strong support was also found throughout the Euctenizidae, including generic relationships within the family and species relationships within the genus *Aptostichus*. As in the Euctenizidae, virtually identical topologies were inferred with high support throughout *Aphonopelma*.

**Conclusions:**

The Spider Probe Kit, the first implementation of AHE methodology in Class Arachnida, holds great promise for gathering the types and quantities of molecular data needed to accelerate an understanding of the spider Tree of Life by providing a mechanism whereby different researchers can confidently and effectively use the same loci for independent projects, yet allowing synthesis of data across independent research groups.

**Electronic supplementary material:**

The online version of this article (doi:10.1186/s12862-016-0769-y) contains supplementary material, which is available to authorized users.

## Background

Spiders (Order Araneae) constitute a diverse radiation of generalist predatory arthropods with over 45,000 nominal species [1] placed among 3,977 genera and 114 families; some estimates suggest the group may comprise as many as 120,000 species [2]. Diversifying since the Devonian (over 380 million years ago) [3, 4], this ancient group plays a dominant predatory role in almost every terrestrial ecosystem. In addition to their remarkable ecological importance, diversity, and abundance, spiders are known for their extraordinary biomolecules like venoms and silks, and have become models for behavioral and evolutionary studies (see reviews in [1, 5]).

The consensus view of spider phylogeny, summarized by Coddington and Levi [6] and Coddington [7], has changed relatively little over the past decade. The Order Araneae is divided into two suborders, the Mesothelae and Opisthothelae. The Mesothelae have a number of unique features including medially positioned spinnerets on the venter of the abdomen, vestiges of the abdominal segmentation common to more ancestral arachnid orders (e.g., scorpions), and are limited to one family (Liphistiidae) found only in Southeast Asia. The Opisthothelae comprises the infraorders Mygalomorphae and Araneomorphae. Mygalomorphae (e.g., trapdoor spiders, tarantulas, funnel web spiders and their relatives) is less diverse nominally (~6 % of the total number of described species) and has retained a number of features considered plesiomorphic among spiders (two pairs of book lungs, production of few, and biomechanically weak, silk types). Within the Araneomorphae (e.g., jumping spiders, wolf spiders, orb-weaving spiders, etc.), the monophyly of the major Haplogynae and Entelegynae clades is weakly to moderately supported on the basis of relatively few morphological features. The Haplogynae all lack a sclerotized epigyne (external female genitalic structure) and their monophyly is weakly based on characters from the chelicerae, palp, and spinnerets. Entelegynes typically possess a more complex reproductive system (a sclerotized epigyne with external copulatory openings), and comprise the vast majority of diversity in spiders. The entelegynes consist of multiple hyperdiverse groups (see [4]), including the Retrolateral Tibial Apophysis (RTA) clade (males possessing a unique morphological character on the palp - nearly half of all described spider taxa); the Dionychans (e.g., wolf, fishing, and jumping spiders) [8]; and the Orbiculariae (the lineage that contains most orb weaving spiders and their relatives) [9].

Despite considerable effort, progress in spider molecular systematics has lagged behind the advances made in other comparable arthropod groups. Unfortunately, this has hindered family-level resolution, classification, and tests of important macroevolutionary hypotheses (e.g., the origin of sticky silks, various web types and hunting strategies). Until recently, only 15 markers (13 of which are independent) had previously been used in spiders for phylogeny inference, very few of which are effective at the species level (Additional file 1: Table S1; for examples of loci and how they were used, see [10–18]). A major problem with the traditional markers is that they have often produced misleading or contradictory results [2]. The poor resolution often seen in traditional markers indicate it will take more than a handful of the “usual suspect” loci (markers that easily amplify and sequence, regardless of their phylogenetic information) [89] to overcome the stochasticity of sequence evolution and incongruence across spider gene trees.

### Phylogenomics

Phylogenomic datasets, enabled by high-throughput sequencing, are transforming systematic biology by allowing researchers to confidently resolve many branches on the Tree of Life (TOL) [19] via the ability to gather vast quantities of genomic data with relative ease. Very recently, phylogenomic analyses based on transcriptome data (e.g., 3, 4) have for the first time been applied to deeper-level spider relationships. These studies clearly indicate relationships among spider families require a major overhaul (e.g., the non-monophyly of orb weavers). The use of hundreds and even thousands of loci identify the non-monophyly of the Orbiculariae and suggest that the orb web is likely plesiomorphic for entelygyne spiders [3]. Subsequently corroborated by Garrison et al. [4], this more recent study also calls into question the placement of the key haplogyne family Leptonetidae, as well as resolution among a number of the RTA clade families, and identifies areas on the phylogeny where putative bursts of diversification have occurred.

Although the use of transcriptome sequencing for phylogenetics has greatly advanced our understanding of relationships, by resolving deeper-level relationships of spiders [4], the generally conserved nature of transcribed protein-coding genes can have limited utility for resolving relationships towards the tips of the Tree of Life. Additionally, RNAseq sample prep and sequencing remains comparatively costly, both financially and in terms of feasibility (i.e., collection and storage - eliminating the ability to obtain samples from natural history collections). Transcriptome data are also dependent on expression patterns of the tissue sampled, can have potential issues with contamination and paralogy, and importantly, large amounts of missing data can be associated with transcriptome sequencing following quality control and assembly as taxon specific loci drop out during the loci filtering process (see [3]). Several recent papers call attention to the negative impact of missing data seen in large phylogenomic (transcriptome-based) datasets [20–23]. Moreover, conflicting phylogenetic signal has also been observed when transcriptome and targeted sequence capture data sets are compared see [24]. Unfortunately, it can be difficult to determine which phylogeny (transcriptome or targeted sequence capture) represents the true evolutionary history of the organisms under investigation. At this time, we simply do not have enough information (i.e., taxon sampling, or an understanding of genome evolution and the true selective pressures underlying the loci used in these analyses). We do know that one potential reason for this conflict is that recombination could be occurring in the loci sequenced from transcriptome data [25, 26], especially in genes with exons separated by long introns, a known issue in spiders (i.e., spider genomes are large with short exons and long introns, reminiscent of mammalian genomes; [27]). When adjacent sites in an alignment reside large distances from each other in the genome, gene tree discordance contributes phylogenetic noise ([28], but see [29]). While phylogenetic inference can be misled by recombination for any nuclear loci, whether obtained through AHE or transcriptomes, it is a particular danger for the types of loci frequently recovered in transcriptome sequencing, and one we attempt to address through the use of Anchored Hybrid Enrichment.

Lemmon et al.’s [30] targeted sequence capture or targeted hybrid enrichment (Anchored Hybrid Enrichment), Faircloth et al.’s [31] highly conserved regions (Ultraconserved Elements), and Li et al.’s [32] protein-coding genes, present alternative targeted sequencing approaches that focus on discovering and utilizing conserved regions throughout the genome. These significant changes in the loci available for phylogenetic inference provide a powerful approach for resolving both deep and shallow relationships across the animal TOL. Rather than sequencing only the expressed portions of a genome, hundreds or thousands of *a priori* loci, deemed putatively phylogenetically-informative, are targeted from across the genome to recover these conserved regions along with their flanking regions, using massively parallel high-throughput sequencing. Whereas the efficacy of these approaches has been demonstrated for vertebrate taxa [24, 30, 33–41], the vast majority of invertebrate taxa lack reference genomes and contain considerable variation in genome size and complexity. For example, spiders exhibit large differences in chromosome number and genome size between and even within families [27, 42], an issue that has slowed the development of genomic resources. Our modified approach (described below) builds upon this previous vertebrate work, but makes available high-throughput targeted sequencing to non-model organisms and groups that lack fully assembled, annotated genomes.

### Anchored Hybrid Enrichment in spiders

Anchored Hybrid Enrichment (AHE) [30] is a relatively new DNA sequencing methodology designed to recover hundreds of unique orthologous loci (i.e., single copy, phylogenetically-informative markers) from across the genome, for resolving both shallow and deep-scale evolutionary relationships within non-model systems. In its first incarnation, assembled, annotated vertebrate genomes were employed to build a scaffold from which probes were designed in conserved anchor regions neighboring variable flanking regions spread throughout the genome, creating a diverse set of probes that include exons, introns, intergenic, and conserved regions of the genome (see [19]). Anchored Hybrid Enrichment aims to increase phylogenetic power by providing access to datasets that allow for the sampling of more loci than what is currently available for most taxonomic groups, allow for an increase in number of taxa sampled (via multiplexing), and include loci informative at varying evolutionary time scales. Multiple factors make AHE an attractive and useful approach for systematists, including, but not limited to: its efficiency in non-model species, the high phylogenetically-informative content of the loci across shallow to deep taxonomic scales, the potentially low levels of missing data, rapid data collection, and cost effectiveness (see [19]).

By generating a genomic dataset that is one order of magnitude larger than those produced by traditional methods, we demonstrate that AHE provides a high-throughput sequencing approach that can be used to answer longstanding questions regarding the relationships and diversification of major spider groups, thereby providing the phylogenetic framework for addressing important evolutionary questions (e.g., the evolution of silk use and the evolution of spinning structures like the cribellum). As proof of concept, we apply AHE across three taxonomic depths within the Spider TOL to infer highly supported phylogenies: deep-level family relationships, with a particular emphasis on the infraorder Mygalomorphae (33 taxa); family and intergeneric relationships spanning the mygalomorph family Euctenizidae (25 taxa); and finally, species-level relationships in the North American tarantula genus *Aphonopelma* (83 taxa).

## Methods

We present a modification of Lemmon et al. [30] that expands AHE for species lacking reference genomes. As described in Lemmon et al. (30; pg. 741), “Fully assembled genomes are not necessarily required for this effort; because probe regions were chosen based on conservation and uniqueness, low-coverage genome sequencing could potentially provide enough reads overlapping with probe regions to facilitate inclusion of underrepresented groups in the probe design.” With this in mind, our aim was to design a set of probes to capture loci that are informative at a diversity of timescales across the evolutionary history of spiders.

Following the original AHE paper [30], a target of ~500 loci was chosen because simulation studies suggest the number of loci needed to resolve a phylogeny can range from tens to hundreds depending on the phylogenetic content of the loci (e.g., population size, and time between speciation events) [43–45]. A dataset of this size should be sufficient to resolve difficult nodes (e.g., short branch lengths relative to population size), as in the case of rapid radiations and recent divergences, while also accounting for deep coalescence, incomplete lineage sorting, and/or hybridization [44–48]. We focused on producing a set of markers that balanced the number of loci to be sequenced per individual, with the need for high sequencing coverage per sample, while at the same time efficiently capturing the anchor and flanking regions needed for informative sequence variation.

### Probe design

Hybrid enrichment involves hybridizing short probe sequences to genomic DNA with subsequent enrichment of those regions of interest prior to sequencing. This approach allows the target loci to be separated from non-target regions of the genome [30]. Because capture efficiency is dependent on the similarity between probe and target, and because our aim was to use the same probe set across a very old taxonomic group, we maximized the chance of success by targeting conserved regions across spider genomic data using long, 120 bp capture probes, with dense tiling. One of the advantages of this approach is that loci present across deep taxonomic groups (e.g., Arthropods) can be targeted using the same approach (e.g., hybrid enrichment), yielding datasets that can later be combined across studies. Because enrichment efficiency depends strongly on the level of sequence divergence between the probe and target sequences, however, an ideal kit will utilize probes derived from sequences that are both taken from the clade of interest and also represent the sequence diversity across the clade. This anchored phylogenomic approach, in which probes representing the diversity of the clade are used in probe design, allows one to target more variable probe regions with higher efficiency. From our experience, the tradeoff between utility and efficiency is optimized when target loci are identified at the level of phylum (or lower); probe kits are then developed for each order, with represented lineages being separated by no more than 200 million years (depending on the level of conservation of the targets).

One additional aspect of hybrid enrichment worth noting is that the robustness of the process to sequence divergence between the target species and the probes allows one to obtain all homologs of the target locus that have at least a moderate level of similarity to the target sequences (e.g. 70 %). This is advantageous for three reasons. First, it helps to ameliorate potential errors in identifying orthology during the probe design process. Second, it allows for one to obtain both copies resulting from unexpected gene duplications occurring in lineages not used to develop the probe set. Lastly, it allows one to collect data efficiently in clades with few or no single copy genes. For example, AHE has recently been used to target a comprehensive set of duplicated genes in Teleosts (Vertebrata, Actinopterygii, Teleostei), which have experienced two whole-genome duplications followed by loss of different genes in different lineages (Stout et al. in press).

We utilize as a starting point a published set of orthologous loci identified using a diverse set of insect taxa. Next, we identify putative arthropod-wide loci by searching for these loci in Arachnids, and then develop an Araneae-specific kit for efficient capture in spiders. More specifically, we began with nucleotide alignments of 4485 1:1 orthologs identified using OrthoMCL by Niehuis et al. ([49, 50]; Supplementary_file_archive_09.tgz) that contain 13 insects: 11 members of Holometabola from five orders (Diptera, Hymenoptera, Lepidoptera, Strepsiptera, and Coleoptera) and 2 non-holometabolous insects from 2 orders (Phthiraptera and Hemiptera). A full list of the species and their higher taxonomy is given in Table A of Additional file 2: Table S2. We then identified for each of the 13 species, corresponding sequences in the 1,478 transcriptome-based 1:1 orthologs identified by Misof et al. [51] using OrthoDB 5. After identifying the loci overlapping between the Niehuis and Misof studies, we selected a preliminary set of target loci by retaining only those Niehuis [49, 50] alignments containing ≥6 taxa and at least one consecutive 120 bp region with >50 % pairwise sequence identity. Sequences for each species were then extracted and saved in separate fasta files (one per species). Exon boundaries were then identified using published genomes and custom scripts that identified matches between the transcript sequences and the genomes using 40-mers (scripts available in our Dryad supplemental materials, doi:10.5061/dryad.5027d) [90].

Together with the alignments, the exon boundaries were used to identify suitable candidate regions (exons) to target using an anchored phylogenomics approach, as described by Lemmon et al. [30]. The following requirements were used to select the 962 preliminary targets: A) the region was at least 150 bp in length; B) the region contained no exon boundaries; and C) the region contained no indel gaps. Details of these targets are given in Table B of Additional file 2: Table S2; alignments can be found in our Dryad supplemental materials [90]. The lengths of these targets ranged from 150 nt to 863 nt (mean = 187 nt), whereas the pairwise sequences similarity ranged from 45 % to 84 % (mean = 66 %).

We scanned the preliminary targets for homologous sequences in transcriptomes from 17 species across all spiders (though heavily biased towards the Mygalomorphae), and the tick *Ixodes* genome (Ixodida, Ixodidae) (Additional file 2: Table S2, Table C), using three divergent insects as references: *Tribolium* (Order Coleoptera), *Mengenilla* (Order Strepsiptera), and *Bombyx* (Order Lepidoptera). Then, for each locus, we used MAFFT (v7.023b with -genafpair and -maxiterate 1000 flags; [52]) to construct an alignment containing the best matching transcript from each of the 18 species (with 55 % match minimum). Loci not containing both *Aliatypus* and *Aphonopelma* (Araneae, Mygalomorphae) were not considered further. In order to ensure good lineage and transcript representation, we also removed loci for which more than 2 of the remaining species had transcript lengths less than 80 % as long as the maximum length of the two representatives. This winnowing process resulted in 225 alignments, with an average length of 854 bases (total 192,333 bp per species).

In order to identify exon boundaries in the transcript alignments, >15x coverage raw genomic reads were obtained for *Aliatypus* and *Aphonopelma* at the Translational Science Laboratory in the College of Medicine at Florida State University (see GenomeSequencingSummary.xlsx for details, Additional file 3: Table S3). Raw genomic reads from each species were mapped to the 225 transcript sequences from the corresponding species and exon boundaries were identified as positions at which reads mapping on each side had poor matches on the opposite side. This process yielded 592 exons.

In addition to transcriptome-based data, we incorporated three additional Araneae lineages for which assembled genomes were available from the i5k Genome Sequencing Initiative ([53]; https://www.hgsc.bcm.edu/arthropods): the black widow (*Latrodectus hesperus*; Lhes.scaffolds.fa; made available on website 12/06/2013), the common house spider (*Parasteatoda tepidariorum*; Ptep01282013.genome.fa; made available on website 03/26/2013), and the brown recluse spider (*Loxosceles reclusa*; Lrec.scaffolds.fa made available on website 01/29/2014). We scanned each genome using references derived from the 592 exon alignments described above, and extracted from the genomes the regions corresponding to each exon (trimmed to match exon boundaries). Note that the more recently published *Stegodyphus* and *Acanthoscurria* genomes [27] were not included in the development of the Spider Probe Kit due to their availability occurring only after the kit was designed.

Spiders possess large, highly repetitive genomes, which present special challenges because repetitive elements can reduce capture efficiency [54–56]. Spider genome sizes have been reported to range from an estimated <1 Gb to ≥11 Gb [27, 57], and our own genomic sequencing of the mygalomorph genera *Aliatypus* and *Aphonopelma* estimates the genome sizes to be 2.5Gb and 16Gb respectively. With that in mind, we checked for high-copy regions (e.g., microsatellites and transposable elements) in the probe region sequences using the three genome-derived references as follows. First, a database was constructed for each species using all 15-mers found in the trimmed probe region alignments for that species. We also added to the database all 15-mers that were 1 bp removed from the observed 15-mers. The three genomes were then exhaustively scanned for the presence of exact matches to these 15-mers, with matches tallied at the corresponding probe region alignment position. Alignment regions containing >100,000 counts in any of the three species were masked to prevent probe tiling across these regions. Probes of 120 bp were tiled uniformly at 4.0x tiling density (for each of the 21 reference species = 18 transcriptomes + 3 genomes). Scripts used for locus selection and design, alignments, final probe region sequences, and the final probe sequences are available as supplemental materials in Dryad [90]. As this process only produced 35,630 of the 57,700 available in an Agilent Sure-Select enrichment kit, we replicated the number of probes for short loci (see Ara1KeyWithReplicates.xlsx for details, Additional file 4: Table S4).

### Sampling for sequencing

The newly developed AHE probe set was employed to evaluate efficacy at three taxonomic depths across the Spider TOL (see Table 1 for list of sequenced taxa). To investigate deep-level family relationships across Araneae, 33 taxa were selected including *Liphistius* (Mesothelae) and lineages throughout the Opisthothelae (both Mygalomorphae and Araneomorphae). The second taxonomic depth investigated intergeneric relationships across the mygalomorph family Euctenizidae, where 25 taxa were sequenced covering all genera, as well as species relationships within the genus *Aptostichus*. The Euctenizidae were rooted with the family Idiopidae, its putative sister lineage [17]. To examine the ability of the probe kit to resolve species-level relationships, we investigated a putative recent radiation into the United States by sequencing 77 individuals within the North American tarantula genus *Aphonopelma*. Provided there was enough material, two specimens of each *CO1* (DNA barcode) species proposed in Hamilton et al. [58] were sequenced; samples encompassed the geographic and genetic breadth of each putative species, including “cryptic species”. Unfortunately, at the time of sequencing, some of these *CO1* species were singletons or lacked good nuclear DNA for sequencing (i.e., old specimens whose tissue was not well preserved for genomic sequencing), so only one specimen represented that species. Six additional specimens of Central American and Caribbean genera (i.e., *Aphonopelma*, *Cyrtopholis*, *Sericopelma*, *Stichoplastoris*, and an unknown putative novel genus from Mexico) were included to test the monophyly of the North American *Aphonopelma*. Furthermore, to evaluate the utility of AHE sequencing on specimens in natural history collections, we extracted and sequenced six specimens in differing states of preservation. Specimens that were designated as old tissue (see Table 1) included taxa that were either ≥10 years old and stored in ≥95 % EtOH in a −20 °C freezer (AUMS_11407, AUMS_11458, MY_2873), taxa ≥10 years old and stored in 80 % EtOH on a shelf in the AUMNH collection (EU_80, MY_3548), or taxa collected ≤10 years old, initially preserved in Isopropyl EtOH, transferred to ≥95 % EtOH and stored in a −80 °C freezer (APH_0948). High-quality genomic DNA (≥1 μg) for all specimens was extracted using a Qiagen DNeasy Blood & Tissue kit, drawn from leg tissue that had been preserved using RNAlater or ≥95 % EtOH and stored in a −80 ° C freezer within the AUMNH cryo-collection, or from the shelves of the AUMNH. DNA concentration was evaluated through agarose gel electrophoresis and spectrophotometry using a NanoDrop ND-1000.

**Table 1:** A list of taxa sequenced for this project, and their taxonomic groupings (A = Araneae; B = Euctenizidae; C = *Aphonopelma*)

Specimen	Family	Genus	Species epithet	Dataset	
MY_2873	Actinopodidae	*Actinopus*	sp.	A	^a^
AUMS_4177	Antrodiaetidae	*Antrodiaetus*	*unicolor*	A	
AUMS_11407	Araneidae	*Verrucosa*	sp. Guatemala	A	^a^
AUMS_719	Atypidae	*Sphodros*	sp.	A	
AUMS_11401	Barychelidae	*Psalistops*	sp. Panama	A	
MY_2635	Ctenizidae	*Hebestatis*	*theventi*	A	
MY_565	Ctenizidae	*Stasimopus*	sp.	A	
AUMS_4566	Ctenizidae	*Ummidia*	sp. North Carolina	A	
MY_515	Cyrtaucheniidae	*Ancylotrypa*	sp.	A	
MY_3399	Cyrtaucheniidae	*Fufius*	sp.	A	
MY_530	Cyrtaucheniidae	*Homostola*	*paradalina*	A	
AUMS_11400	Dipluridae (?)	*Linothele* (?)	sp. Panama	A	
MY_2485	Euctenizidae	*Apomastus*	sp.	B	
MY_2519	Euctenizidae	*Aptostichus*	*aguacaliente*	B	
MY_3630	Euctenizidae	*Aptostichus*	*angelinajolieae*	B	
MY_0741	Euctenizidae	*Aptostichus*	*atomarius*	B	
MY_3029	Euctenizidae	*Aptostichus*	*barackobamai*	B	
MY_2521	Euctenizidae	*Aptostichus*	*cahuilla*	B	
MY_0730	Euctenizidae	*Aptostichus*	*dantrippi*	B	
MY_2279	Euctenizidae	*Aptostichus*	*edwardabbeyi*	B	
MY_0265	Euctenizidae	*Aptostichus*	*hedinorum*	B	
MY_2496	Euctenizidae	*Aptostichus*	*hesperus*	B	
MY_2512	Euctenizidae	*Aptostichus*	*icenoglei*	B	
MY_3771	Euctenizidae	*Aptostichus*	*madera*	B	
MY_3524	Euctenizidae	*Aptostichus*	*miwok*	B	
MY_3081	Euctenizidae	*Aptostichus*	*simus*	A,B	
AUMS_11458	Euctenizidae	*Aptostichus*	sp. Baja - MX	B	^a^
MY_3486	Euctenizidae	*Aptostichus*	*stanfordianus*	B	
MY_3492	Euctenizidae	*Aptostichus*	*stephencolberti*	B	
AUMS_11404	Euctenizidae	*Entychides*	sp. Arizona	B	
MY_3548	Euctenizidae	*Entychides*	sp. Mexico	B	^a^
EU_80	Euctenizidae	*Eucteniza*	sp.	B	^a^
AUMS_053	Euctenizidae	Moss Landing gen. nov.	sp.	B	
AUMS_081	Euctenizidae	*Myrmekiaphila*	*tigris*	B	
MY_272	Euctenizidae	*Neoapachella*	sp.	B	
MY_3688	Euctenizidae	*Promyrmekiaphila*	sp.	B	
MY_2049	Hexathelidae	*Atrax*	sp.	A	
MY_2045	Hexathelidae	*Bymainiella*	sp.	A	
AUMS_156	Hypochilidae	*Hypochilis*	sp.	A	
AUMS_6747	Idiopidae	*Idiops*	sp. Namibia	A,B	
AUMS_5743	Liphistiidae	*Liphistius*	sp.	A	
AUMS_11467	Lycosidae	*Schizocosa*	*crassipes*	A	
AUMS_5112	Lycosidae	*Schizocosa*	*duplex*	A	
AUMS_5615	Lycosidae	*Schizocosa*	*ocreata*	A	
AUMS_5605	Lycosidae	*Schizocosa*	*rovneri*	A	
salt-a	Lycosidae	*Schizocosa*	*saltatrix*	A	
AUMS_5604	Lycosidae	*Schizocosa*	*stridulans*	A	
AUMS_0548	Mecicobothriidae	*Hexura*	sp.	A	
MY_2138	Migidae	*Heteromigas*	sp.	A	
MY_2096	Nemesiidae	*Chenistonia*	sp.	A	
MY_2139	Nemesiidae	*Ixamatus*	sp.	A	
MY_2094	Nemesiidae	*Kiama*	sp.	A	
AUMS_6720	Nemesiidae	*Pionothele*	sp. Namibia	A	
MY_2092	Nemesiidae	*Stanwellia*	*hoggi*	A	
AUMS_6256	Paratropididae	*Paratropis*	sp. Columbia	A	
APH_0856	Theraphosidae	*Aphonopelma*	*anax*	C	
APH_3121	Theraphosidae	*Aphonopelma*	*anax*	C	
APH_0871	Theraphosidae	*Aphonopelma*	*anax*	C	
APH_0889	Theraphosidae	*Aphonopelma*	*anax*	C	
APH_0554	Theraphosidae	*Aphonopelma*	*armada*	C	
APH_0922	Theraphosidae	*Aphonopelma*	*armada*	C	
APH_1478	Theraphosidae	*Aphonopelma*	*atomicum*	C	
APH_1479	Theraphosidae	*Aphonopelma*	*atomicum*	C	
APH_3001	Theraphosidae	*Aphonopelma*	*belindae*	C	
APH_1438	Theraphosidae	*Aphonopelma*	*catalina*	C	
APH_1602	Theraphosidae	*Aphonopelma*	*catalina*	C	
APH_0174	Theraphosidae	*Aphonopelma*	*chalcodes*	C	
APH_0954	Theraphosidae	*Aphonopelma*	*chalcodes*	C	
APH_1003	Theraphosidae	*Aphonopelma*	*chalcodes*	C	
APH_0049	Theraphosidae	*Aphonopelma*	*chalcodes*	C	
APH_3128	Theraphosidae	*Aphonopelma*	*chalcodes*	C	
APH_3191	Theraphosidae	*Aphonopelma*	*chiricahua*	C	
APH_1009	Theraphosidae	*Aphonopelma*	*eutylenum*	C	
APH_3143	Theraphosidae	*Aphonopelma*	*eutylenum*	C	
APH_0850	Theraphosidae	*Aphonopelma*	*gabeli*	C	
APH_3126	Theraphosidae	*Aphonopelma*	*gabeli*	C	
APH_0645	Theraphosidae	*Aphonopelma*	*hentzi*	A,C	
APH_0934	Theraphosidae	*Aphonopelma*	*hentzi*	C	
APH_1063	Theraphosidae	*Aphonopelma*	*hentzi*	C	
APH_0586	Theraphosidae	*Aphonopelma*	*hentzi*	C	
APH_3118	Theraphosidae	*Aphonopelma*	*icenoglei*	C	
APH_0756	Theraphosidae	*Aphonopelma*	*icenoglei*	C	
APH_2000	Theraphosidae	*Aphonopelma*	*iodius*	C	
APH_2029	Theraphosidae	*Aphonopelma*	*iodius*	C	
APH_2016	Theraphosidae	*Aphonopelma*	*iodius*	C	
APH_0006	Theraphosidae	*Aphonopelma*	*iodius*	C	
APH_2020	Theraphosidae	*Aphonopelma*	*iodius*	C	
APH_3103	Theraphosidae	*Aphonopelma*	*iodius*	C	
APH_1075	Theraphosidae	*Aphonopelma*	*iodius*	C	
APH_1078	Theraphosidae	*Aphonopelma*	*iodius*	C	
APH_2013	Theraphosidae	*Aphonopelma*	*iodius*	C	
APH_0997	Theraphosidae	*Aphonopelma*	*iodius*	C	
APH_1004	Theraphosidae	*Aphonopelma*	*iodius*	C	
APH_2032	Theraphosidae	*Aphonopelma*	*johnnycashi*	C	
APH_2008	Theraphosidae	*Aphonopelma*	*johnnycashi*	C	
APH_3094	Theraphosidae	*Aphonopelma*	*johnnycashi*	C	
APH_1007	Theraphosidae	*Aphonopelma*	*joshua*	C	
APH_1498	Theraphosidae	*Aphonopelma*	*joshua*	C	
APH_3177	Theraphosidae	*Aphonopelma*	*madera*	C	
APH_0136	Theraphosidae	*Aphonopelma*	*madera*	C	
APH_1618	Theraphosidae	*Aphonopelma*	*mareki*	C	
APH_3161	Theraphosidae	*Aphonopelma*	*mareki*	C	
APH_0297	Theraphosidae	*Aphonopelma*	*mareki*	C	
APH_0772	Theraphosidae	*Aphonopelma*	*marxi*	C	
APH_1535	Theraphosidae	*Aphonopelma*	*marxi*	C	
APH_0890	Theraphosidae	*Aphonopelma*	*moderatum*	C	
APH_0930	Theraphosidae	*Aphonopelma*	*moderatum*	C	
APH_0892	Theraphosidae	*Aphonopelma*	*moderatum*	C	
APH_0894	Theraphosidae	*Aphonopelma*	*moderatum*	C	
APH_0948	Theraphosidae	*Aphonopelma*	*moellendorfi*	C	^a^
APH_0754	Theraphosidae	*Aphonopelma*	*mojave*	C	
APH_3101	Theraphosidae	*Aphonopelma*	*mojave*	C	
APH_3166	Theraphosidae	*Aphonopelma*	*paloma*	C	
APH_3190	Theraphosidae	*Aphonopelma*	*paloma*	C	
APH_3178	Theraphosidae	*Aphonopelma*	*parvum*	C	
APH_3187	Theraphosidae	*Aphonopelma*	*parvum*	C	
APH_0683	Theraphosidae	*Aphonopelma*	*peloncillo*	C	
APH_0723	Theraphosidae	*Aphonopelma*	*peloncillo*	C	
APH_0350	Theraphosidae	*Aphonopelma*	*prenticei*	C	
APH_0352	Theraphosidae	*Aphonopelma*	*prenticei*	C	
APH_3176	Theraphosidae	*Aphonopelma*	*saguaro*	C	
APH_0622	Theraphosidae	*Aphonopelma*	sp. nov. 1	C	
APH_0880	Theraphosidae	*Aphonopelma*	sp. nov. 2	C	
APH_3012	Theraphosidae	*Aphonopelma*	sp. burica	C	
APH_1023	Theraphosidae	*Aphonopelma*	*steindachneri*	C	
APH_3096	Theraphosidae	*Aphonopelma*	*steindachneri*	C	
APH_1443	Theraphosidae	*Aphonopelma*	*superstitionense*	C	
APH_0185	Theraphosidae	*Aphonopelma*	*vorhiesi*	C	
APH_0186	Theraphosidae	*Aphonopelma*	*vorhiesi*	C	
APH_0717	Theraphosidae	*Aphonopelma*	*vorhiesi*	C	
APH_0674	Theraphosidae	*Aphonopelma*	*vorhiesi*	C	
APH_3188	Theraphosidae	*Aphonopelma*	*vorhiesi*	C	
APH_3132	Theraphosidae	*Aphonopelma*	*xwalxwal*	C	
APH_3134	Theraphosidae	*Aphonopelma*	*xwalxwal*	C	
APH_3056	Theraphosidae	*Cyrtopholis*	*portoricae*	C	
APH_3004	Theraphosidae	*Sericopelma*	sp. Panama	C	
AUMS_10968	Theraphosidae	*Stichoplastoris*	sp. Costa Rica	C	
APH_3021	Theraphosidae	unknown genus	sp. Mexico dwarf	C	

^a^denotes a tissue as “old” (see text for explanation)

### Library preparation, enrichment, and sequencing

Anchored Hybrid Enrichment data were collected through the Center for Anchored Phylogenomics at Florida State University (www.anchoredphylogeny.com) following the general methods of Lemmon et al. [30] and Prum et al. [59]. Briefly, each genomic DNA sample was sonicated to a fragment size of ~175-325 bp using a Covaris E220 Focused-ultrasonicator with Covaris microTUBES. Subsequently, library preparation and indexing were performed on a Beckman-Coulter Biomek FXp liquid-handling robot following a protocol modified from Meyer and Kircher [60]. One important modification is a size-selection step after blunt-end repair using SPRIselect beads (Beckman-Coulter Inc.; 0.9x ratio of bead to sample volume). Indexed samples were then pooled at equal quantities (approximately 16 samples per pool), and enrichments were performed on each multi-sample pool using an Agilent Custom SureSelect kit (Agilent Technologies), described herein as the Spider Probe Kit, that contained the probes designed for AHE loci from the spider genomic data detailed above. After enrichment, each set of reactions were pooled in equal quantities for sequencing on three PE150 Illumina HiSeq2500 lanes. Sequencing was performed in the Translational Science Laboratory in the College of Medicine at Florida State University.

### Data processing

**Fig. 1:**
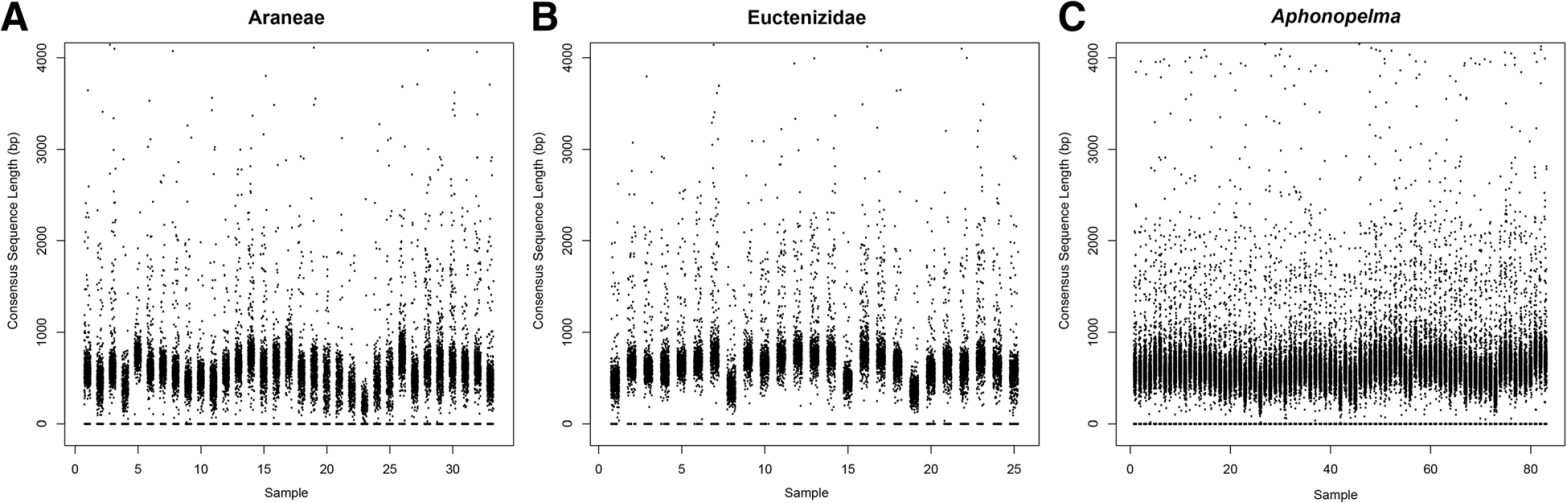
Assemblies for each taxon group and specimen (**a** = Araneae, **b** = Euctenizidae, **c** = *Aphonopelma*), used to produce the homologous sequence sets. Each point represents one consensus sequence, with the length (bp) on the y-axis and the sequences grouped by sample on the x-axis

Reads were filtered for quality using Illumina’s Casava software with a high chastity filter. Read length and accuracy was improved by merging overlapping reads following Rokyta et al. [61]. This process also trimmed and removed adapters. Merged reads and non-overlapping read pairs were used downstream. All reads were assembled into contigs following Prum et al. [59], but using references derived from the *Ixodes*, *Hypochilus*, *Aphonopelma*, and *Aliatypus* sequences used for probe design. The assembler produces separate contigs for gene copies differing by more than 5 % sequence divergence. To reduce errors caused by low-level indexing errors during sequencing, contigs were then filtered by removing those derived from fewer than 35 reads. Supplemental file P0073_AssemblySummary_Summary.xlsx (Additional file 5: Table S5) provides a summary of the sequence data collected and resulting assemblies. Figure 1 illustrates these assemblies, for each taxon group and specimen. Each point represents one consensus sequence, with the length (bp) on the y-axis and the sequences grouped by sample on the x-axis. The majority of consensus sequences are shorter than 1000 bp, though there is a small group of loci per individual that are greater. The exceptionally long assemblies are due to messy ends (e.g. microsats) and are discarded during the trimming steps. Due to thresholds designed to minimize missing data, the *autotrimmer* removes these jagged ends of the alignments when creating consensus sequences.

Homologous sequence sets were produced by grouping filtered consensus sequences across individuals by target locus. Orthologous groups were then determined for each target locus following Prum et al. [59]: A) a pairwise-distance measure was computed for pairs of homologs, with distance being computed as the percentage of 20-mers observed in the two sequences that were found in both sequences. In order to accommodate variable third codon positions in exonic regions, we included 20-mers comprised bases found every third position in addition to 20-mers from contiguous; B) a neighbor-joining clustering algorithm was then used to cluster the consensus sequences into orthologous sets, with at most one sequence per species in each orthologous set. The algorithm is as follows: A) compute pairwise 20-mer distance for all homologs as described above; B) identify the two homologous sequences from different individuals with the minimum distance; C) group the two identified sequences in a cluster; D) repeat steps A-C, treating each cluster as a sequence by using the average distance between the cluster and other clusters/sequence when assessing distance. For duplications occurring before the ancestor of the taxonomic group being analyzed, the method efficiently divides the homologs into two or more orthologous sets. For duplications occurring within the taxonomic group under investigation, the method tends to produce a single cluster containing one sequence per taxon and a second cluster containing sequences from those taxa that are descendants from the ancestor in which the duplication occurred. Cases in which orthology is difficult result in several clusters each containing a fraction of the taxa. In order to improve this situation and to minimize the effects of missing data, clusters containing fewer than (75 %) of the species were removed from downstream processing. This approach to orthology assessment is particularly well suited to data collected by AHE for two reasons. First it allows the utilization of noncoding sequences (e.g. flanks) that tend to be more variable and thus useful for identifying orthology at shallower taxonomic scales. Second it allows one to utilize taxa from remote portions of the Tree of Life that are underrepresented in databases such as OrthoDB [62].

Sequences in each orthologous set were aligned using MAFFT v7.023b [52] with --genafpair and --maxiterate 1000 flags. In order to remove poorly aligned regions, raw alignments were then trimmed and masked following Prum et al. [59], with the following adjustments: sites with >70 % similarity were identified as good, 20 bp regions containing <10 good sites were masked, and sites with fewer than 10 unmasked bases were removed from the alignment.

### Phylogenetic inference

For each taxonomic dataset, a concatenated supermatrix of all loci was constructed to infer relationships using Maximum Likelihood (ML) phylogenetic inference in RAxML v8.1.9 [63, 64]. Because RAxML is limited in the choice of models of sequence evolution that can be chosen, we used the (GTR + Γ) model. Based on evaluating the most appropriate model of sequence evolution on a subsample of the Spider Probe Kit loci using jModelTest v2.1.7 [65], the GTR + Γ model was found to only slightly over-parameterize most loci. Parameters for the concatenated RAxML analyses were based on 1000 random addition sequence replicates (RAS), with branch support values computed via 1000 non-parametric bootstrap replicates, and partitions were set for each locus.

With the knowledge that a single gene tree almost certainly will not reflect the true evolutionary history of a lineage [66], we attempted to infer the species tree for each of our three taxonomic depth investigations. Modern species tree inference methods that jointly estimate gene trees and the species tree under the multispecies coalescent model (MSC) are unable to feasibly utilize phylogenomic datasets of this size (both computationally and temporally, e.g., *BEAST). Due to this limitation, we inferred phylogenies for each individual locus using RAxML, under the same guidelines as above with subsequent species tree estimation using ASTRAL II v4.7.8 [67], from all individual unrooted gene trees (and bootstrap replicates). ASTRAL, a gene-tree method based in coalescent theory, attempts to find the species tree that agrees with the largest number of quartet trees, from a set of gene trees, under a “quasi” multispecies coalescent approach. Quartet-based supertree methods combine many quartet trees into a single and coherent tree, over the complete set of taxa (see [68]). ASTRAL has been shown to be statistically consistent under the multi-species coalescent model, is thought to be a fast and accurate alternative to other “shortcut” or “two-step” coalescent methods like MP-EST [69], and importantly, ASTRAL performs consistently in the anomaly zone ([29, 70–72]; Warnow unpublished – see http://tandy.cs.illinois.edu). An advantage of inferring phylogenies from multiple loci is that if relationships consistently emerge across individual gene trees, confidence can be taken from this phylogenetic resolution.

Additionally, investigations into simulated and empirical datasets have shown that, if there is strong incongruence between loci, concatenated supermatrices are likely to return incorrect trees that are strongly supported [70, 71, 73]. For deep phylogenetic problems, the utility of a coalescence approach has been debated (see [29, 74, 75]). The idea that shortcut coalescence methods (e.g., STEM, STAR, and MP-EST) perform poorly when individual gene trees are inferred and subsequently used to sort deep coalescences among gene trees has been proposed by Gatesy and Springer [74]. Mirarab et al. [69] have shown that concatenation can be more accurate than phylogenetic inference under the multi-species coalescent, if low amounts of incomplete lineage sorting are present, yet under biologically realistic conditions, coalescent-based methods can be more accurate than concatenation [29], conflicting with Gatesy and Springer [74]. A potential explanation for this conflict and/or lack of resolution is that present phylogenetic methods cannot adequately model the complex processes of molecular evolution inherent in such large data sets (see [76]). Thus we feel it is important to analyze datasets, such as these herein, using both a combined supermatrix and species tree phylogenetic inference. An approach such as this provides opportunities to more thoroughly detect and investigate gene tree discordance or hidden signal throughout the dataset. Because of these fundamental differences, we chose to infer phylogenies using both the concatenated supermatrix and MSC approaches, and evaluate agreement or discordance between them. All investigations were carried out on the CASIC HPC at Auburn University and the FSU Center for Anchored Phylogenomics.

## Results & discussion

### Probe design & sequencing

**Fig. 2:**
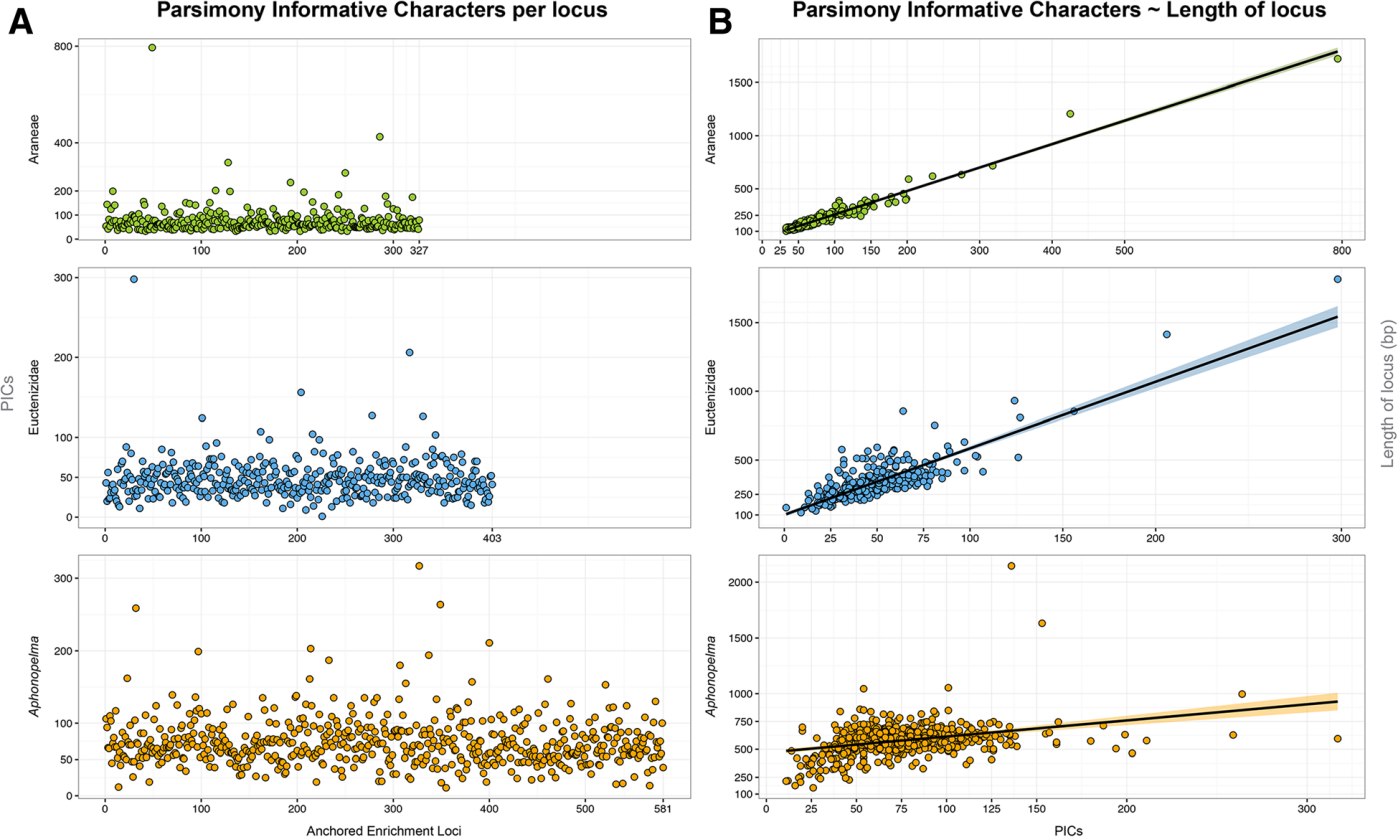
Parsimony Informative Characters (PICs) for each taxon dataset and each locus. **a** represents the number of PICs in each locus. **b** represents the correlation between the length of a locus and the number of PICs in that locus

**Fig. 3:**
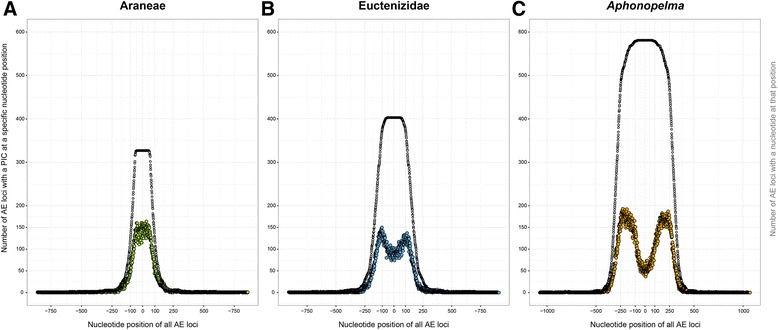
The distribution of Parsimony Informative Characters (PICs) for the three taxon groupings (**a** = Araneae, **b** = Euctenizidae, **c** = *Aphonopelma*) and their Anchored Hybrid Enrichment (AHE) loci. The zero position is the center of the anchor sequence for all loci. Colored dots indicate how many total loci had at least one PIC at that nucleotide position (*left y-axis*). Grey dots indicate the distribution of locus length by plotting the number of loci (*right y-axis*) with any nucleotide at that position. The figures are plotted on the same y-axis scale to more easily compare the levels of variation across the taxonomic scales

The Spider AHE Probe Kit, comprises a total of 585 orthologous target loci chosen on the basis of conservation and uniqueness, capable of constituting a dataset with over 400,000 bp. Based on the divergence estimates reported in Bond et al. [3] and Garrison et al. [4], the taxa used as genomic models to develop the Spider Probe Kit encompass an estimated time span of >300 mya, the widest implementation of targeted hybrid enrichment to date. Following recovery and analysis, shorter loci correspond to genomic regions that are less variable and appear better at recovering deep to medium nodes, whereas longer loci are highly variable and work well for resolving more shallow nodes (in particular, species relationships). The length of the loci utilized in this analysis range from 101 bp to 2146 bp, with parsimony informative characters (PICs) ranging from 1 to 794 per locus (Figs. 2 & 3). The numbers of PICs across the taxonomic groupings are significantly correlated with length (bp) in all of the datasets (each *p* < 2e-16; Araneae Adjusted R-squared = 0.9575, Euctenizidae Adjusted R-squared = 0.7064, *Aphonopelma* Adjusted R-squared = 0.1179).

As seen in Lemmon et al. [30] and herein, a smaller number of loci are captured from deeper taxonomic depths, (e.g., the 581 loci in the *Aphonopelma* dataset decreases to 327 in the Araneae dataset; see Figs. 2 & 3). The biological reality is that not all loci should be expected to capture, enrich, and sequence properly for all individuals; loci are lost due to a number of possibilities, including deep divergence between outgroup and ingroup, sequence change in the probe region, poor quality DNA, library preparation failure, poor quality of sequencing reads, and issues during assembly and orthology assessment. Though the number of captured orthologs shared across our sampled taxa decreases as evolutionary distances among the lineages increases, a decline in number of loci does not correspond with a loss in relative informativeness, as evidenced by robust support throughout our phylogenies. We now know a simple count of the numbers of loci used for phylogeny inference should not be compared to the numbers of loci one can obtain from other sequencing approaches (e.g., UCEs or transcriptome data). The argument that the use of more loci in an analysis provides a better outcome is not necessarily the truest case for robust phylogeny inference; an ideal approach is to utilize *a priori* loci deemed phylogenetically-informative for the particular question [77]. Although the spider probe kit provides good enrichment efficiency (1 %-15 % of reads mapping to target loci), given the large genome size, deep taxonomic depth, and variable sample quality, an increased representation of Araneomorphae would increase the capture efficiency in that clade. Additional sequencing information (e.g., number of reads recovered for each sample) can be viewed in P0073_AssemblySummary_Summary.xlsx (Additional file 5: Table S5). The Illumina sequencing reads and assembled sequences have been deposited in the Dryad data repository (http://datadryad.org, doi:10.5061/dryad.5027d).

### Order Araneae

**Fig. 4:**
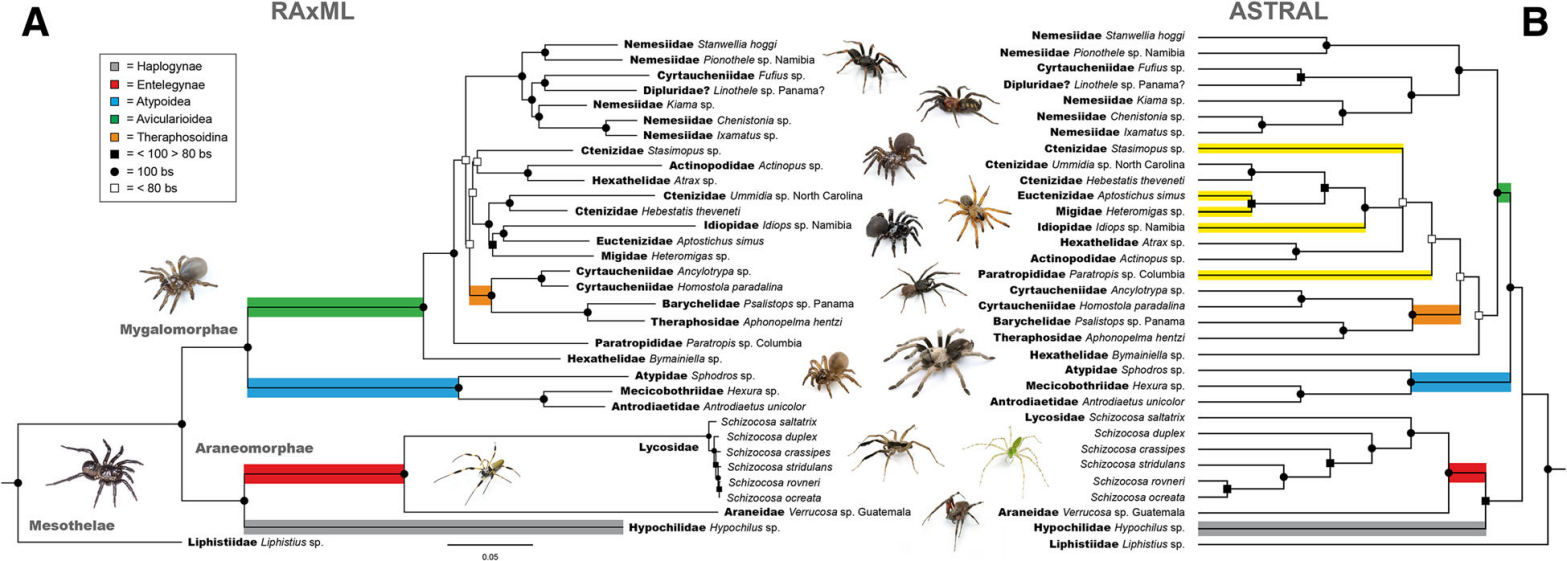
**a** - Maximum Likelihood analysis of the 33 Araneae taxa concatenated supermatrix. The dataset comprises 327 loci and 67,870 bp. Black circles denote 100 % bootstrap support; black squares denote bootstrap support between 99-80 %; white squares denote bootstrap support less than 80 %. **b** - ASTRAL species tree inference based on the Maximum Likelihood inferred individual gene trees from the 327 loci Araneae dataset. ASTRAL analyzes unrooted gene trees; tree was subsequently rooted at the branch leading to the Liphistiidae outgroup. ASTRAL node support values = support based on the RAxML bootstrap support from all trees and all loci. Our preference of the supermatrix inferred phylogeny is likely the more appropriate evaluation of the data at this timescale. Lineages with a different placement between the two outcomes are indicated in yellow. Inset key and colors denote certain historical taxonomic groupings recovered with our sampling. Photographs illustrate a generalized spider lineage corresponding to that region of the phylogeny

Following hybrid enrichment, sequencing, and implementation of the AHE bioinformatics pipeline (outlined above), a dataset of 327 loci comprising 67,870 bp for 33 OTUs was selected for phylogenetic inference and evaluation of the efficacy of this methodology to recover Araneae relationships. This dataset contains 30,340 variable sites, 25,871 informative sites, and 16 % total missing characters. Of the 327 loci, an average of 29 taxa were represented in each locus. The length of the individual alignments ranged between 101 bp and 1721 bp, with a mean of 207 bp, and the number of PICs per locus range from 33–794 (Figs. 2 & 3a). *Liphistius* was used to root the phylogeny [3]. Analyses of the concatenated supermatrix and ASTRAL species tree estimation were largely congruent, with high bootstrap support among most nodes. The supermatrix phylogeny recovers all three major spider lineages with high bootstrap support, the Mesothelae, Mygalomorphae, and Araneomorphae. Due to phylogenetic methodology restrictions at this time (see [29, 69, 74, 75]), our preference of the supermatrix inferred phylogeny is likely the more appropriate evaluation of the data at this timescale (Fig. 4a). Lineages with a different placement between the two outcomes are indicated in yellow on the phylogeny (Fig. 4b). Within this dataset, sampling is sparse among araneomorphs at this time, reflecting our initial emphasis on mygalomorph taxa - a relatively well-sampled Mygalomorphae clade consisting of 14 out of the 16 families (missing the Dipluridae and Microstigmatidae). Within the Araneomorphae, the Entelegynae clade is represented by the orb-weaver genus *Verrucosa* (Araneidae) and the wolf spider genus *Schizocosa* (Lycosidae). In both the supermatrix and ASTRAL analyses, the entelegyne clade is monophyletic and sister to the Haplogynae (represented by *Hypochilus*). Additionally, species-level relationships within *Schizocosa* are highly supported (>95 bootstrap support (bs)).

Within the Mygalomorphae, sampling is not sufficient enough to allow for a direct comparison with the findings of Bond et al. [3], however major, long-hypothesized lineages such as the Atypoidea (Atypidae, Antrodiaetidae, and Mecicobothriidae), Avicularioidea (all other mygalomorph families), and Theraphosoidina are well supported in both inference approaches, (Figs. 2 and 3). There is high support for a number of nodes in both the supermatrix and species tree analyses. Importantly, these include: A) within the Avicularioidea, there is one highly supported, monophyletic clade consisting of Nemesiidae lineages (*Stanwellia*, *Pionothele*, *Kiama*, *Chenistonia*, and *Ixamatus*), the cyrtaucheniid genus *Fufius*, and a specimen from Panama that was intended as a representative of the family Dipluridae, identified morphologically as the genus *Linothele*, however its placement in the phylogeny clearly indicates that it is most likely a nemesiid; B) a sister group relationship between Actinopodidae (*Actinopus*) and the Hexathelidae genus *Atrax*; C) a monophyletic sister group relationship between the North American ctenizid genera *Ummidia* and *Hebestatis*); D) a highly supported, monophyletic Theraphosoidina that includes a monophyletic clade of the cyrtaucheniid genera *Ancylotrypa* and *Homostola*, sister to a monophyletic clade of Barychelidae (*Psalistops*) and Theraphosidae (*Aphonopelma*). Unfortunately, across both analyses there is a lack of resolution at the medium nodes within the Mygalomorphae. Though largely congruent between the two phylogenies, there is conflicting placement of the families Actinopodidae, Ctenizidae, Euctenizidae, Hexathelidae, Idiopidae, Migidae, and Paratropididae at those intermediate nodes. Significant disagreement occurs between the supermatrix and species tree analyses in the placement of the Paratropididae (*Paratropis*), the ctenizid genus *Stasimopus*, and the hexathelid *Bymainiella*. The supermatrix analysis indicates high support for the placement of *Bymainiella* (Hexathelidae) and *Paratropis* (Paratropididae) as the sister lineages to the vast majority of mygalomorph diversity, consistent with Bond et al. [3], whereas in the species tree paratropidids are the sister group to the clade that includes ctenizids, euctenizids, idiopids, and other taxa.

Important comparisons with Bond et al. [3] that continue to emphasize the problems in mygalomorph classification include: both analyses corroborate the elevation of euctenizines to the family level, though the sister group to Euctenizidae (supermatrix = Idiopidae, species tree = Migidae) (Fig. 4) remains unresolved, without increased sampling; *Actinopus* and *Atrax* are robustly supported as sister, though their placement in the phylogeny differs greatly; and lastly, both analyses provide evidence of at least three polyphyletic families (Ctenizidae, Cyrtaucheniidae, and Hexathelidae), and indicate the Ctenizidae are polyphyletic with respect to the placement of the genus *Stasimopus*.

The low support within certain medium-depth nodes and our limited sampling precludes a definitive understanding of mygalomorph families (e.g., the Crassitarsae and whether the Nemesiidae are sister to the Theraphosoidina, as well as the placement of the families Dipluridae and Microstigmatidae - both critically important to understanding mygalomorph evolution). While increasing taxon sampling will likely increase node support for most of the tree [77], given the evolutionary depth of this clade, it is more likely that removing saturated sites from future datasets will aid in improving support values, especially in the middle to deep regions of the phylogeny.

### Euctenizidae

**Fig. 5:**
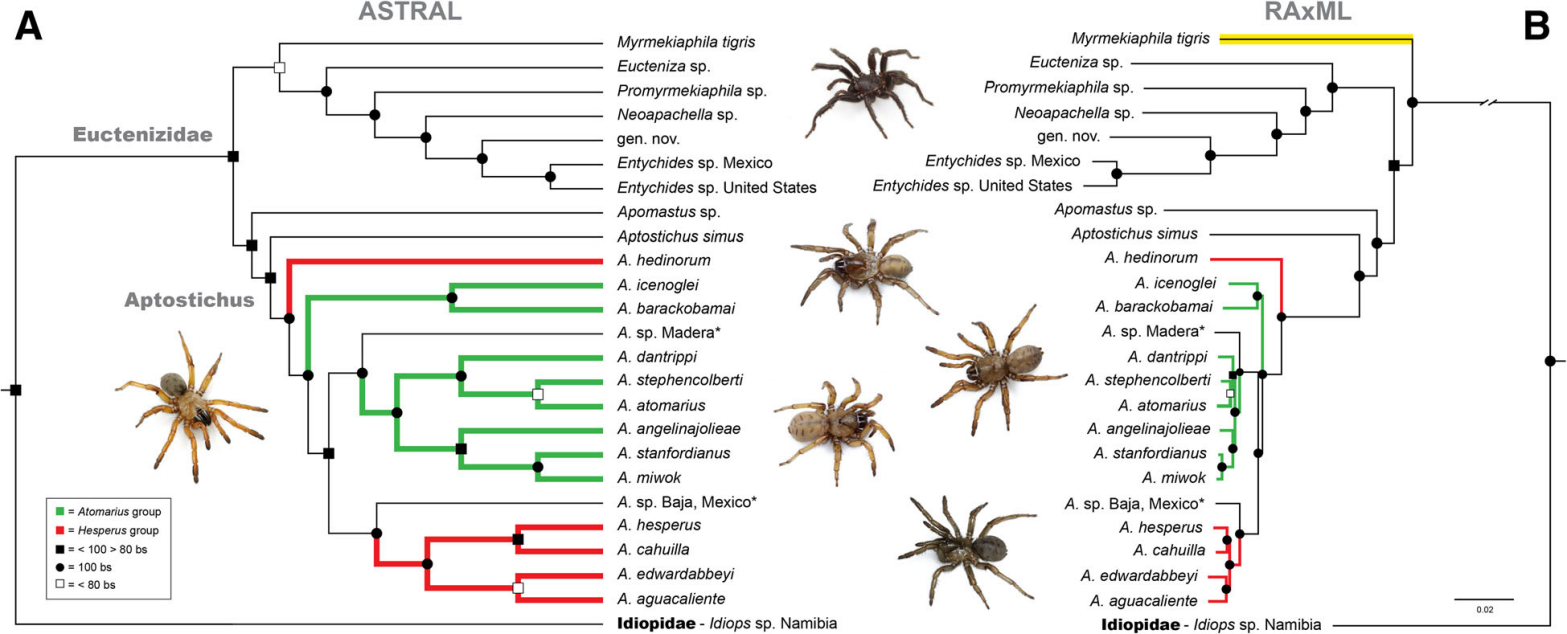
**a** - ASTRAL species tree inference based on the Maximum Likelihood inferred individual gene trees from the 25 taxa Euctenizidae family/genus level dataset, comprising 403 loci and 133,614 bp. ASTRAL analyzes unrooted gene trees; tree was subsequently rooted at the branch leading to the Idiopidae outgroup. ASTRAL node support values = support based on the RAxML bootstrap support from all trees and all loci. **b** - Maximum Likelihood analysis of the Euctenizidae concatenated supermatrix. Black circles denote 100 % bootstrap support; black squares denote bootstrap support between 99-80 %; white squares denote bootstrap support less than 80 %. The species tree inference is likely the more appropriate evaluation of the data at this timescale. Inset key and colors denote certain historical taxonomic groupings recovered with our sampling. The non-monophyly of the morphological *Atomarius* and *Hesperus* species groups is identified. Novel taxa are denoted by an asterisk. Photographs illustrate a generalized spider lineage corresponding to that region of the phylogeny

The 25 sequenced taxa include family and inter-generic relationships spanning the mygalomorph family Euctenizidae (Table 1). Following hybrid enrichment, sequencing, and implementation of the AHE bioinformatics pipeline, a dataset of 403 loci comprising 133,614 bp was selected for phylogenetic inference. The dataset contains 42,046 variable sites, 22,548 informative sites, and 12.5 % total missing characters. Of the 403 loci, an average of 24 taxa were represented in each locus. The length of the individual alignments ranged between 117 bp and 1817 bp, with a mean of 331 bp, and the number of PICs range from 1–298 (Figs. 2 & 3b). The family Idiopidae, a putative closely related sister lineage based on preliminary data and Bond et al. [17], was used to root the phylogeny, a result not confirmed when more taxa were included (see above). The supermatrix and species tree analyses produced virtually identical topologies within the Euctenizidae, with high bootstrap support among nearly all nodes, and producing strongly supported generic relationships within the family and species relationships within the genus *Aptostichus* (Fig. 5). All deep and intermediate node support was high (>80 bs). As discussed above, due to methodological restrictions at this time (see [29, 69, 74, 75]), the species tree inference is likely the more appropriate evaluation of the data at this timescale (Fig. 5a).

Comparison of the phylogenies herein with the *Aptostichus* morphological phylogeny proposed by Bond [78] identifies disagreement with respect to species groups and species-level relationships. Additionally, relationships between the other euctenizid genera included in Bond [78] are drastically different (*Neoapachella*,(*Eucteniza*,(*Entychides*,(*Promyrmekiaphila*,(*Myrmekiaphila*,(*Apomastus*,*Aptostichus*)))))), when compared to our inferred relationships (Fig. 5a). In Bond [78], *Myrmekiaphila* is hypothesized as being closely related to *Aptostichus*, significantly different from our results. Within *Aptostichus*, the non-monophyly of the *Hesperus* group and the *Atomarius* group is strongly supported (Fig. 5). The *Hesperus* group is rendered polyphyletic due to the removal of *A. hedinorum* and placing it as the sister lineage to all other sampled *Aptostichus*, except for the basal *A. simus* lineage. The tree also identifies a paraphyletic *Atomarius* group, with the placement of a monophyletic clade from the morphological *Hesperus* group inside the *Atomarius* clade (Fig. 5).

### *Aphonopelma*

**Fig. 6:**
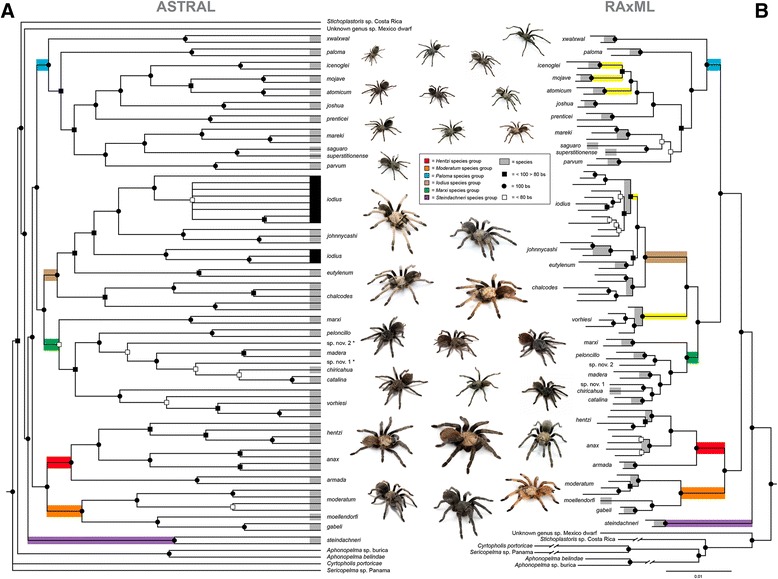
**a** - ASTRAL species tree inference based on the Maximum Likelihood inferred individual gene trees from the 83 taxa Euctenizidae family/genus level dataset, comprising 581 loci and 334,436 bp. ASTRAL analyzes unrooted gene trees; tree was subsequently rooted at the branch leading to the non-*Aphonopelma* outgroup taxa. ASTRAL node support values = support based on the RAxML bootstrap support from all trees and all loci. **b** - Maximum Likelihood analysis of the *Aphonopelma* concatenated supermatrix. Black circles denote 100 % bootstrap support; black squares denote bootstrap support between 99-80 %; white squares denote bootstrap support less than 80 %. The species tree inference is likely the more appropriate evaluation of the data at this timescale. Inset key and colors denote taxonomic groupings recovered with our sampling. All genealogically exclusive species [79] are identified with a grey bar; *A. iodius*, a paraphyletic species as presently defined, is identified by the black boxes. Novel taxa are denoted by an asterisk. Lineages with a different placement between the two outcomes are indicated in yellow. Photographs illustrate the *Aphonopelma* species corresponding to that region of the phylogeny

Following hybrid enrichment, sequencing, and implementation of the AHE bioinformatics pipeline, a dataset of 581 loci comprising 334,436 bp for 83 OTUs was selected for phylogenetic inference. The dataset contains 79,912 variable sites, 44,342 informative sites, and 7.3 % total missing characters. Of the 581 loci, an average of 80 taxa were represented in each locus. The length of the individual alignments ranged between 156 bp and 2146 bp, with a mean of 575 bp, and the number of PICs range from 11–317 (Figs. 2 & 3c). To evaluate the monophyly of the genus, Central American taxa (*Aphonopelma belindae* (APH_3001), *A*. sp. *burica* (APH_3012), *Sericopelma* sp. (APH_3004), *Stichoplastoris* sp. (AUMS_10968)), a putative novel genus of miniature tarantula from Mexico (APH_3021), and a genus from the Caribbean (*Cyrtopholis portoricae*, APH_3056) were included. Both analyses indicate that *Aphonopelma*, as presently defined, is not monophyletic, with all other genera (i.e., morphologically divergent) sampled rendering the genus paraphyletic. As in Euctenizidae, the supermatrix and species tree estimation produced virtually identical topologies with high bootstrap support throughout (Fig. 6). The phylogeny inferred through the species tree approach is likely the more appropriate evaluation of the data at this timescale, and thus the species tree was our preferred topology (Fig. 6a). Lineages with a different placement between the two outcomes are indicated in yellow on the phylogeny (Fig. 6b).

There are six major morphologically distinct lineages identified in the phylogeny, as defined by Hamilton et al. [79]: the *Hentzi* species group, the *Moderatum* species group, the *Marxi* species group (from the Madrean Archipelago/Sky Islands region of southeastern Arizona and southwestern New Mexico), the *Iodius* species group, a group of miniature *Aphonopelma* (the *Paloma* species group), and the *Steindachneri* species group. In the species tree analysis, all of these major lineages, except the *Marxi* species group (59 bs), are highly supported (100 bs) and monophyletic (Fig. 6a). All 28 of the recently revised species in Hamilton et al. [79] are strongly supported as representing genealogically exclusive, independently evolving lineages, with one exception, *Aphonopelma iodius*. In the species tree analysis, all species are monophyletic and genealogically exclusive, with high support (≥90 bs), except *A. iodius* - paraphyletic with respect to *A. johnnycashi*, itself a highly supported monophyletic clade that is morphologically and geographically distinct. Though monophyletic in the supermatrix analysis with high support (95 bs), *A. iodius* is a known problematic species (see [79]) that has likely experienced high levels of gene flow between populations and rapid expansion and growth in the relative recent geologic past. The only major difference in topology seen across both analyses corresponds to the placement of *A. marxi* and *A. vorhiesi*. The supermatrix dataset strongly supports (100 bs) the placement of *A. marxi* as a member of the *Marxi* species group, but excludes *A. vorhiesi*, which is placed as the sister lineage (100 bs) to the *Iodius* species group - a grouping similarly seen in the *CO1* data (see Hamilton et al. [58, 79]), and likely caused by mitochondrial introgression. In the *CO1* dataset, a sister relationship is inferred between *A. vorhiesi* and *A. chalcodes* in the face of radically divergent morphology and phenotype. *Aphonopelma vorhiesi* is a medium size black spider and *A. chalcodes* is large bodied with contrasting light and dark. Nothing in our knowledge of these two species suggests this sister relationship to be accurate (see [79]), but we do know that these two species are sympatric throughout much of SE Arizona. In comparison, the species tree places both *A. marxi* and *A. vorhiesi* within the *Sky Islands* species group. Additionally, this outcome highlights the dangers of using a concatenated supermatrix approach to infer phylogenetic relationships at the species level, particularly if there are nodes that fall within the anomaly zone [29].

Lastly, when we compare the mitochondrial (*CO1*) species outlined by Hamilton et al. [58] to the AHE species tree, a number of these previously putative species hypotheses correspond to lineages that were considered “cryptic species”, yet when viewed in the light of the AHE data are no longer distinct (e.g., sp. nov. A - *A. vorhiesi* cryptic, sp. nov. B - *A. vorhiesi* cryptic, sp. nov. F - *A. peloncillo* cryptic, sp. *moderatum* nov. sp. *hentzi* nov. 1). These findings illuminate how deep mitochondrial divergence and introgression have obscured our understanding of the true evolutionary history of these lineages - a phenomenon seen more often now as nuclear loci are more easily gathered (see [80]). This outcome is not surprising given the large number of papers showing how the use of a single locus, like mtDNA data, to delimit species can be misleading (e.g., [81, 82]).

## Conclusions

By specifically targeting 585 phylogenetically informative loci across the Order Araneae, the AHE Spider Probe Kit provides an order of magnitude larger dataset than any previously generated by traditional targeted sequencing approaches. Anchored Hybrid Enrichment delivers phylogenetic utility at both deep and shallow taxonomic depths thereby providing a much needed set of molecular markers that can be used to address evolutionary questions at multiple hierarchical levels. Though our research is not the first to implement this type of targeted sequencing in invertebrates (e.g., studies in divergent groups throughout Class Insecta are beginning to be published (Diptera - [83]; Hymenoptera - [84, 85]; and Lepidoptera - Breinholt and Kawahara [91])), it is the first implementation of this methodology in Class Arachnida. While the initial cost for the development of the Spider Probe Kit was high (owing primarily to the large genome sizes of mygalomorphs and the subsequent need for deeper sequencing coverage for the genome scans), costs for the kit, extraction, library preparation, and sequencing continue to decrease, making this approach suitable for large-scale projects or collaborations between researchers. Future users will find that the amount and quality of data recovered is remarkable and the cost per specimen low (for detailed cost information see 19, 30, and www.anchoredphylogeny.com).

We know the use of AHE, or any phylogenomic approach, is not a panacea and that certain nodes in the Tree of Life may be beyond resolution [86–88]. The development of the Spider Probe Kit holds great promise for gathering the types and quantities of molecular data needed to understand the evolution of spiders, including a mechanism whereby researchers can confidently and effectively use the same loci, allowing for future meta-analyses to better understand the spider Tree of Life. Not only will this and future datasets be informative to our understanding of spider evolution, the deep arthropod-wide markers included in the probe design allows the Order Araneae to be included in future research that seeks to understand the deeper Arachnida, Chelicerata, and Arthropoda Tree of Life relationships.

Finally, it is important to note that a major benefit of this approach allows specimens from natural history collections to be sequenced, provided DNA can be retrieved. All of our test specimens (see Methods and Table 1) captured a moderate to exceptional number of loci. This finding opens many new avenues of investigation by allowing for the placement of extinct taxa into a phylogeny, or examining evolutionary response over time to an environmental pressure. Although our initial probe kit development was biased towards mygalomorphs, due to the inclusion of a large number mygalomorph transcriptomes and the two mygalomorph genome scans, a new version of the Spider Probe Kit (v.2) has recently been developed and includes increased genomic scans of araneomorph taxa as well as araneomorph transcriptomes in order to allow the v.2 capture probe set to consistently recover a higher number of loci across all spider taxa under investigation.

## Additional files

Additional file 1: Table S1. The 13 “usual suspect” molecular markers used throughout spiders, previous to the usage of next-generation sequencing for phylogenomics. nuDNA = genomic DNA, mtDNA = mitochondrial DNA (CSV 112 bytes)
Additional file 2: Table S2. Table A - A full list of the species and their higher taxonomy; Table B - Details of the 962 preliminary targets; Table C - Arachnid genomic data used in development of the Anchored Hybrid Enrichment Spider Probe Kit v.1. (XLSX 128 kb)
Additional file 3: Table S3. GenomeSequencingSummary. Summary of the genomic reads obtained for *Aliatypus* and *Aphonopelma*. (XLSX 50 kb)
Additional file 4: Table S4. Ara1_Key_withReplicates. Number of Agilent Sure-Select enrichment kit probes per locus in the Anchored Hybrid Enrichment Spider Probe Kit v.1. (XLSX 1027 kb)
Additional file 5: Table S5. P0073_AssemblySummary. Summary of the sequence data collected and resulting assemblies. (XLSX 80 kb)
